# Pulmonary dermoid cyst mimicking hydatid cyst: a case report with literature review

**DOI:** 10.1093/jscr/rjag941

**Published:** 2026-10-19

**Authors:** Fahmi H Kakamad, Harem K Ahmed, Amanj K Hamad, Ari M Abdullah, Ahmed H Ahmed, Rebaz M Ali, Harun Amanj Ahmed, Mohammed Namiq Amin, Mohammed Abdullah Othman

**Affiliations:** Department of Thoracic and Vascular Surgery, Smart Health Tower, Madam Mitterrand Street, Sulaymaniyah 46001, Iraq; Department of Clinical Sciences, College of Medicine, University of Sulaimani, Madam Mitterrand Street, Sulaymaniyah 46001, Iraq; Kscien Organization for Scientific Research (Middle East Office), Hamdi Street, Sulaymaniyah 46001, Iraq; Department of Pulmonology, Smart Health Tower, Madam Mitterrand Street, Sulaymaniyah 46001, Iraq; Department of Pulmonology, Smart Health Tower, Madam Mitterrand Street, Sulaymaniyah 46001, Iraq; Department of Pathology, Smart Health Tower, Madam Mitterrand Street, Sulaymaniyah 46001, Iraq; Department of Pathology, Sulaymaniyah Teaching Hospital, Zanko Street, Sulaymaniyah 46001, Iraq; Department of Radiology, Smart Health Tower, Madam Mitterrand Street, Sulaymaniyah 46001, Iraq; Department of Oncology, Hiwa Cancer Hospital, Shorsh Street, Sulaymaniyah 46001, Iraq; Department of Oncology, Smart Health Tower, Madam Mitterrand Street, Sulaymaniyah 46001, Iraq; Department of Head & Neck Surgery, Smart Health Tower, Madam Mitterrand Street, Sulaymaniyah 46001, Iraq; Department of Scientific Affairs, Smart Health Tower, Madam Mitterrand Street, Sulaymaniyah 46001, Iraq; Student Research Committee, Kermanshah University of Medical Sciences, Kermanshah 6714415153, Iran; Department of Emergency Medicine, Smart Health Tower, Madam Mitterrand Street, Sulaymaniyah 46001, Iraq

**Keywords:** pulmonary teratoma, dermoid cyst, hydatid cyst, cystic lung lesion, video-assisted thoracoscopic surgery

## Abstract

Intrapulmonary dermoid cyst (mature cystic teratoma) is rare and may mimic common cystic lung diseases, particularly pulmonary hydatid disease. A 26-year-old woman presented with dyspnea and left-sided chest, shoulder, and neck pain. Computed tomography demonstrated a large complex cystic lesion in the left upper lobe with gas locules and associated pleural and pericardial effusions, initially suggesting a hydatid cyst. Video-assisted thoracoscopic excision revealed hair and heterogeneous cyst contents, and histopathology showed mature tissues from multiple germ layers with xanthogranulomatous inflammation, confirming a mature teratoma. The patient remained well at the 3-month follow-up. A focused review of eight reported cases of intrapulmonary teratoma showed heterogeneous presentations and recurrent preoperative diagnostic uncertainty. Intrapulmonary teratoma may present as a complex cystic lung lesion and can be mistaken for a hydatid cyst or a lung abscess.

## Introduction

Germ cell tumors originate from primordial germ cells that fail to complete their embryonic migration toward the gonadal ridge; although the gonads remain the principal site of origin, extragonadal tumors account for only about 1%–5% of all cases, with the anterior mediastinum representing their most frequent location outside the gonads [1]. Among mediastinal germ cell tumors, mature teratomas—composed of well-differentiated tissue derived from more than one of the three germ layers—are the most common benign subtype, whereas the term “dermoid cyst” is classically reserved for lesions built almost exclusively from ectodermal derivatives such as skin, hair, and sebaceous glands, although the two terms are often used interchangeably in clinical practice [1, 2].

While intrathoracic teratomas arise predominantly within the mediastinum, an intrapulmonary origin—confined entirely to the lung parenchyma without any mediastinal or gonadal connection—is exceedingly rare and accounts for only a small fraction of reported thoracic cases [3, 4]. Histologically, mature lesions may contain skin appendages, cartilage, bone, and respiratory, gastrointestinal, or pancreatic tissue, and their prominent cystic component can radiologically simulate a loculated pleural or pericardial effusion, a feature that further complicates preoperative diagnosis [3, 4].

Owing to their nonspecific clinical and radiological presentation, intrapulmonary teratomas are frequently mistaken preoperatively for other cystic or cavitary lung lesions, such as pulmonary hydatid disease [4]. The overlapping imaging appearance of the two entities, both of which may present as large, thick-walled cystic masses, has previously led to misdiagnosis and unwarranted antiparasitic treatment before histopathological confirmation was obtained [4].

This report presents a case of lung dermoid cyst with pericardial and pleural effusion, mimicking a hydatid cyst. This study has been written in line with the CaReL guidelines, and the credibility of the references has been verified [5, 6].

## Case report

### Patient information

A 26-year-old woman presented with shortness of breath and left-sided chest, shoulder, and neck pain for a few days. She reported no associated fever or rigors. Her past medical and surgical history was unremarkable.

### Clinical findings

On examination, the patient was hemodynamically stable with normal vital signs. Respiratory examination revealed decreased air entry over the left hemithorax, with no added sounds.

### Diagnostic assessment

The white blood cell count was 13.6 × 10^9^/L (reference range, 4–11 × 10^9^/l), and the remaining complete blood count parameters were within normal limits. Computed tomography (CT) of the chest demonstrated a large (9 × 9 × 7.7 cm) complex cystic lesion in the left upper lobe with internal necrosis and gas locules, suspected to be a hydatid cyst, accompanied by a mild left pleural effusion, a mild pericardial effusion, and localized atelectasis (Fig. 1).

**Figure 1:**
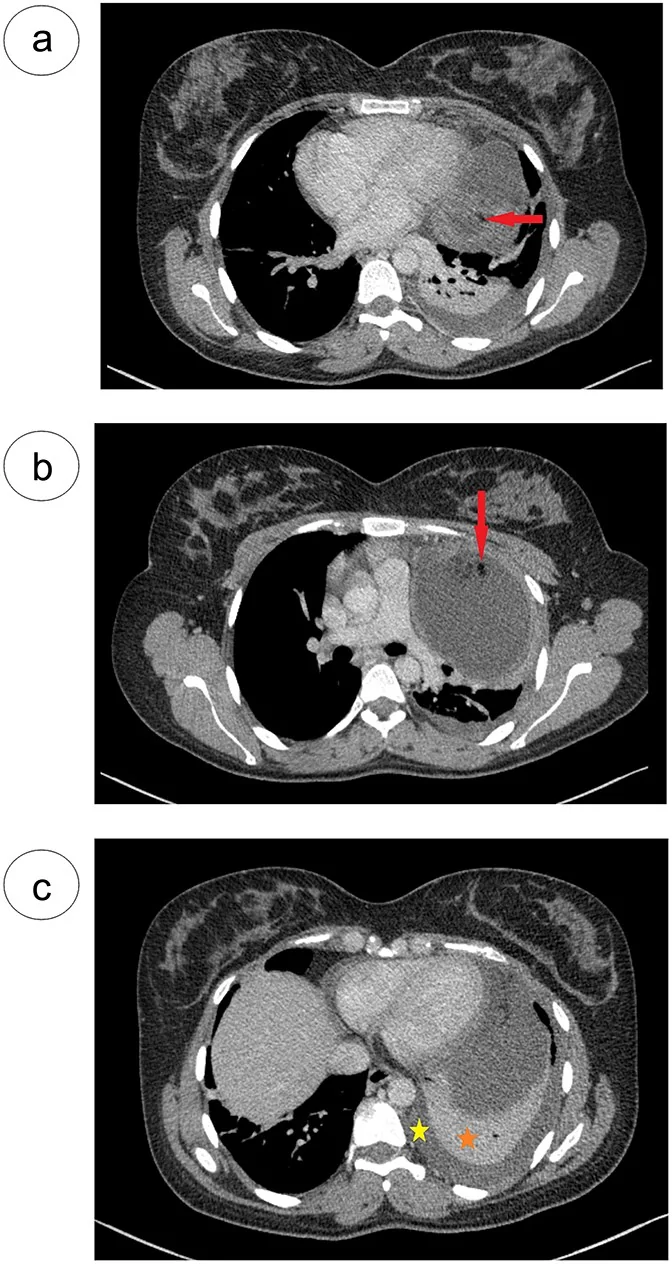
Chest CT scan showing a large complex cystic lesion with internal necrosis in the left upper lung lobe. (a) Fat spots at the lower part (arrow). (b) Left upper lobe large thick enhancing wall with spots of fat within (red arrow). (c) Pleural effusion (yellow asterisk) and lung atelectasis (orange asterisk).

### Therapeutic intervention

The patient underwent video-assisted thoracoscopic surgery. Exploration of the thoracic cavity revealed a loculated, pus-like pleural effusion, which was evacuated and sent for culture and sensitivity testing. A cystic lesion was identified in the left upper lobe. On opening the cyst, hair, stone-like material, and purulent content were encountered. The cyst was evacuated, and the cyst wall was excised (Fig. 2). Hemostasis was secured, cryoablation was performed, a chest tube was inserted, and the wound was closed. Histopathological examination confirmed a benign mature teratoma with xanthogranulomatous inflammation. Microscopically, the specimen showed a fibrous cyst wall lined by squamous, columnar, and intestinal mucosa, and contained mature elements, including skin appendages, cartilage, pancreatic acini, and bone, together with a heavy inflammatory infiltrate and giant cell reactions (Fig. 3).

**Figure 2:**
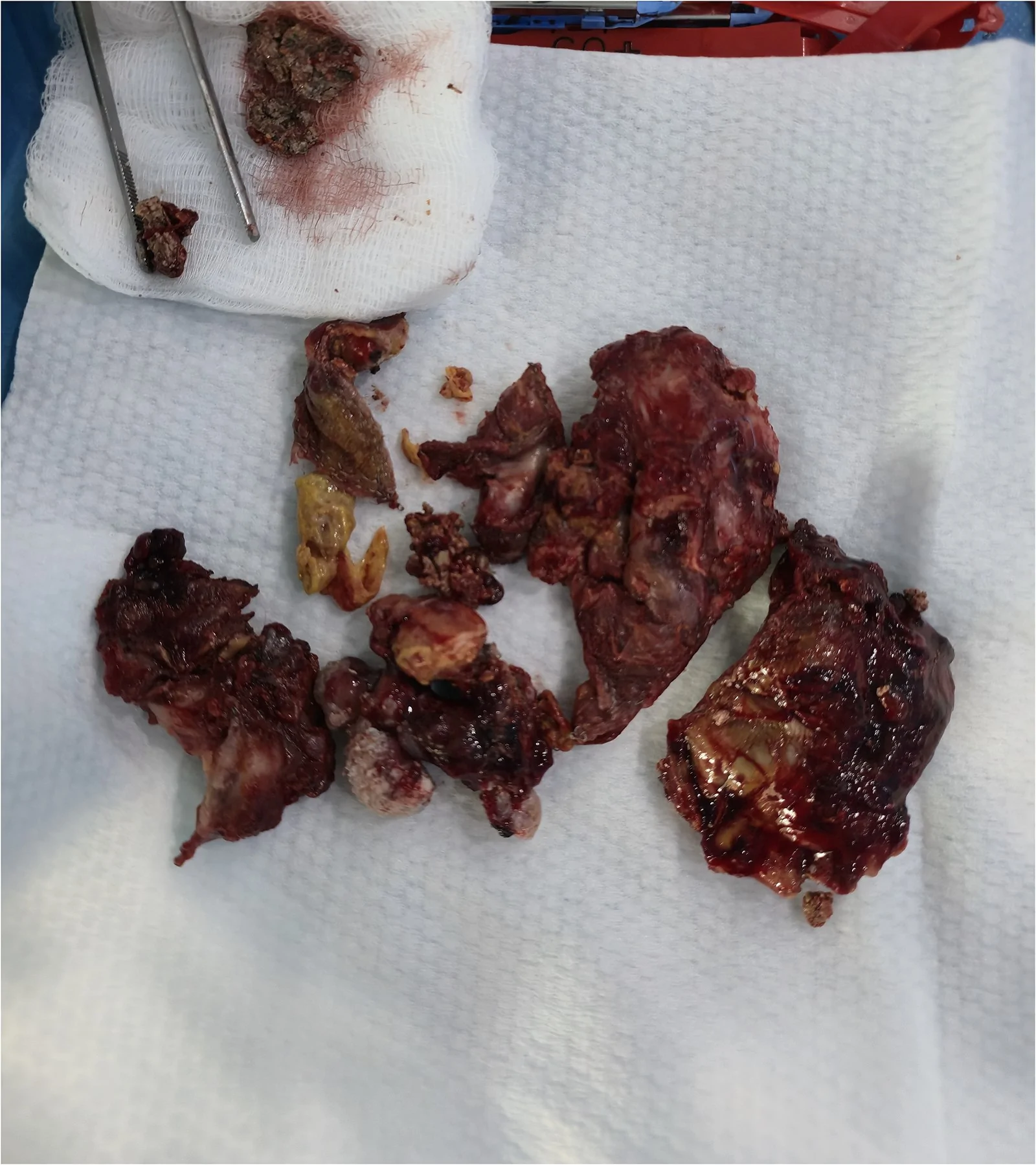
Intraoperative view showing the excised cyst wall containing hair and purulent material.

**Figure 3:**
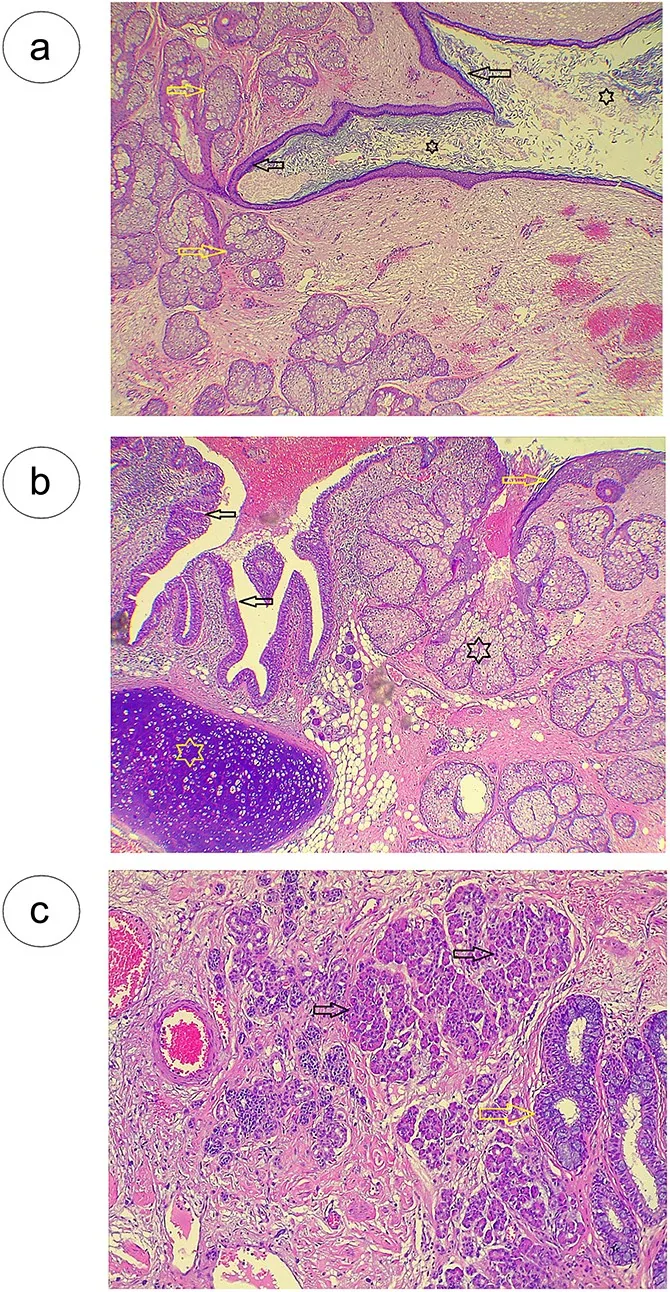
Histopathological views showing a benign mature teratoma with a fibrous cyst wall and mixed inflammatory infiltration. (a) Cystic area lined by benign stratified squamous epithelium, containing keratinous material in the lumen and sebaceous glands in the cyst wall. Hematoxylin and eosin stain, ×4. (b) Ciliated columnar and benign stratified squamous epithelial linings, a sebaceous gland, and a nodule of hyaline cartilage. Hematoxylin and eosin stain, ×4. (c) Pancreatic acini and bland-looking glands lined by goblet cells. Hematoxylin and eosin stain, ×10.

### Follow-up and outcome

Postoperatively, the patient recovered uneventfully, with resolution of her symptoms. The patient was discharged in stable condition, and outpatient follow-up was arranged. At the three-month follow-up, she reported only mild pain at the operative site without fever, and her complete blood count and C-reactive protein level were within normal limits.

## Discussion

Intrapulmonary teratoma is one of the rarest primary neoplasms of the lung, and its diagnosis is seldom established before operation because its clinical and radiological features overlap with those of far more common cystic and cavitary lung diseases [7–9]. Affected patients are typically symptomatic as a result of compression of, or communication with, adjacent bronchovascular structures, and most often report chest pain, cough, hemoptysis, and dyspnea [7–9]. Trichoptysis, although highly specific, is uncommon and has been reported in approximately 13% of cases [7, 8]. Cross-sectional imaging is central to the workup, as a heterogeneous intrapulmonary mass containing fat, fluid, soft tissue, and calcification is highly suggestive; however, the definitive diagnosis generally rests on histopathological examination of the resected specimen [7, 9, 12]. Complete surgical excision is both diagnostic and therapeutic and is generally associated with an excellent prognosis in mature lesions [7, 8, 13]. In the present case, the diagnosis was likewise unsuspected before surgery, was established only after histopathological examination of the excised cyst, and was followed by an uneventful recovery after complete resection.

To contextualize our findings, eight published cases of intrapulmonary teratoma presenting as a cystic lung lesion were reviewed [4, 8–14] (Table 1). Because of the small number of cases, several unreported variables, and the absence of any formal statistical testing, these figures are descriptive only and should not be read as prevalence estimates or as statistically generalizable. The sexes were evenly represented (four women and four men). The reported sex distribution has been inconsistent, ranging from a slight female preponderance to no clear predilection [7, 11, 12]; the near-equal ratio here is in keeping with the latter. Among the seven patients with a numerically reported age, the mean age was 32 years (range, 13–47), and one further patient was described only as a teenager. Our 26-year-old woman fell within this age range. Lesions were left-sided in five of eight cases, and all involved the upper lobe, as in our patient; however, right upper-, middle-, and lower-lobe lesions were reported each in a case [8, 10, 11], so location alone cannot reliably exclude the diagnosis. A hydatid cyst was suspected or included in the preoperative differential diagnosis in three of eight cases [4, 10, 11], as in our patient. Surgical management consisted of lobectomy in five of eight cases, wedge or local resection in two, and pneumonectomy in one. All eight specimens were mature teratomas without malignant transformation, and postoperative recovery was favorable in the cases with reported outcomes, with no recurrence during the available follow-up.

**Table 1:** Summary of eight reported cases of intrapulmonary teratoma/lung dermoid cyst

Author/year	Country	Age/sex	Main clinical presentation	Duration before diagnosis	Lung side	Lobe/segment	Size (cm)	Initial radiologic diagnosis	Key diagnostic modality	Type of surgery	Histopathology	Outcome/follow-up
Mardani *et al*., 2020 [4]	Iran	27/F	Progressive dyspnea, chest pain, non-productive cough, chills, fever, orthopnea	2 weeks (episodic for years)	Left	LUL	13 × 11 × 4	Empyema; hydatid cyst vs lung abscess	Postoperative histopathology	Thoracoscopy converted to posterolateral thoracotomy with left upper lobectomy	Mature cystic teratoma (pancreatic, mucinous, and respiratory epithelium with sebaceous glands, adipose tissue, smooth muscle, and cartilage)	Uneventful; discharged day 8; no recurrence at 6 years
Bawazir *et al*., 2019 [8]	Saudi Arabia	35/F	Dry cough	3 weeks	Right	Right upper lobe (anterior segment)	6.0 × 5.0 × 2.5	Para-pericardial/anterior mediastinal mass	Postoperative histopathology	Thoracoscopic wedge resection	Mature teratoma (containing soft yellow/cheese-like material)	Chest tube removed on postoperative day 2; discharged on postoperative day 3 in good condition
Boonplod *et al*., 2024 [9]	Thailand	47/F	Hemoptysis, intermittent dyspnea (no fever/weight loss)	5 months	Left	LUL	7.4 × 3.7	Germ cell tumor; CT also suggested possible lung cancer; MDCT favored intrapulmonary teratoma based on fat, calcification, and soft-tissue density	MDCT (fat, calcification, soft tissue); confirmed by histopathology	Left thoracotomy + left upper lobectomy	Mature teratoma (squamous epithelium with sebaceous glands, adipose tissue, intestinal epithelium, and pancreatic tissue)	No recurrence at 3 months
Ismavel and Miriam, 2022 [10]	India	Teenage/F	Massive hemoptysis	Not reported	Right	Right middle lobe	Not reported	Pulmonary hydatid cyst	Postoperative histopathology	Exploratory thoracotomy with partial excision (initial); right middle lobectomy for recurrence 4 years later	Mature teratoma	Asymptomatic with no recurrence 16 years after definitive lobectomy (20 years after the initial surgery)
Ram *et al*., 2019 [11]	India	26/M	Cough and hemoptysis; previous hemoptysis with pneumothorax history	N/R	Right	Right lower lobe	13.8 × 14.8 × 14	Pulmonary hydatid cyst, CT showed water-lily-like appearance	Postoperative histopathology	Right posterolateral thoracotomy with right lower lobectomy	Mature intrapulmonary teratoma (ectodermal, mesodermal, and endodermal derivatives)	Lung expanded postoperatively; on follow-up for 24 months; AFP and β-hCG normal
Jiménez García and Compén Chang, 2018 [12]	Peru	13/M	Cough, expectoration, and recurrent hemoptysis; no fever, chest pain, dyspnea, weight loss, or trichoptysis	Started from an unknown period, with exacerbation before outpatient consultation.	Left	LUL (lingula)	8.9 × 5.0 × 1.1	Heterogeneous intrapulmonary tumor with fluid, fat, soft-tissue, and calcium/calcification components.	Postoperative histopathology	Left upper lobectomy	Mature pulmonary teratoma	N/R
Estevez-Cerda *et al*., 2022 [13]	Mexico	37/M	Intermittent productive cough progressing to constant productive cough, exertional then resting dyspnea; green fetid sputum; no hemoptysis	1 year	Left	LUL	8.4 × 5.1 × 4.8	Presumptive aspergilloma	Postoperative histopathology.	Left posterior thoracotomy with total left pneumonectomy	Entirely intrapulmonary mature teratoma/mature intrabronchial teratoma with skin, sebaceous glands, sweat glands, hair follicles, smooth muscle, intestinal glands, pancreatic tissue, and cartilage; no immature tissue	Discharged after 12 days of uneventful recovery; asymptomatic at 1-year follow-up with preserved pulmonary function
Karlic *et al*., 2023 [14]	USA	40/M	Mild exertional dyspnea; restrictive lung disease on PFTs	1.5 years	Left	LUL (pleural + anterior mediastinal extension)	N/R	Left mediastinal mass (mature teratoma) obstructing LV/RV outflow	Chest CT + pulmonary function tests; confirmed by histopathology	En bloc resection + thymectomy + LUL wedge resection	Benign mature teratoma	Uncomplicated postoperative recovery; benign pathology; no additional oncologic therapy required/reported

Data were extracted verbatim from the primary publications, and the pooled figures reported in the text are descriptive only. A hydatid cyst appeared in the preoperative differential diagnosis in 3 of the 8 cases (cases 1, 4, and 5). One lesion (case 8), although intrapulmonary in origin, showed pleural and anterior-mediastinal extension. F, female; M, male; N/R, not reported; CT, computed tomography; MDCT, multidetector CT; PFT, pulmonary function test; LUL, left upper lobe; LV/RV, left/right ventricle.

Other initial impressions included aspergilloma [13] and germ-cell tumor or lung cancer, the latter reclassified as intrapulmonary teratoma after multidetector CT [9]. The initial impression therefore depends heavily on the imaging pattern and on the locally prevalent differential diagnosis. Although all lesions summarized in Table 1 were mature and benign, malignant elements have been reported in pulmonary teratomas; complete resection and thorough histopathological examination therefore remain appropriate [7, 10].

This case makes two specific points, one radiological and one practical. Radiologically, the presence of macroscopic fat within a complex cystic pulmonary lesion, particularly when accompanied by calcification or soft-tissue components [7, 9], may favor a mature teratoma over pulmonary hydatid disease; despite that, misdiagnosis still cannot be excluded. In our patient, macroscopic fat was identified on CT and was, in retrospect, the most useful preoperative clue, whereas firm stone-like material was encountered intraoperatively and mature osseous tissue was identified histologically. The lesson is that a deliberate search for macroscopic fat should form part of the routine assessment of any atypical, thick-walled cystic lung lesion, particularly where hydatid disease is endemic, because it meaningfully shifts the differential toward a teratoma. This signal can nevertheless be masked: in our patient, the coexisting pleural and pericardial effusion, internal gas locules, and superimposed suppurative and xanthogranulomatous inflammation closely simulated an infected hydatid cyst, a lung abscess, or an empyema. Practically, when a teratoma is plausible, an upfront surgical approach may be both diagnostic and curative and avoids the pitfalls of presuming hydatid disease, namely unnecessary antiparasitic therapy and a percutaneous approach to a lesion mistaken for a parasitic cyst. Trichoptysis, although essentially pathognomonic, is rare and was absent in our patient, so its absence does not exclude the diagnosis.

This study has several limitations. It describes a single case, and the accompanying literature review was drawn from a small number of previously published reports; several variables were unreported, the pooled figures are therefore descriptive and hypothesis-generating rather than statistically robust or generalizable, and no inferential testing was performed. Reporting heterogeneity and probable publication bias further constrain interpretation. The definition of “intrapulmonary” also varies across reports; one included lesion, although intrapulmonary in origin, showed pleural and anterior-mediastinal extension [14], underscoring the difficulty of strict anatomical classification. Finally, only short-term (three-month) follow-up of the present patient is currently available.

Intrapulmonary teratoma may present as a complex cystic lung lesion and can be mistaken for a hydatid cyst or a lung abscess before operation. This case suggests that a mature teratoma should be considered among the differential diagnoses of an atypical cystic lung lesion, particularly when imaging is inconclusive.

## Data Availability

All clinical data relevant to this case are included in the article. Additional anonymized information is available from the corresponding author on reasonable request, subject to institutional and patient-confidentiality requirements.
